# Extraction and Study of Hypoglycemic Constituents from *Myrica rubra* Pomace

**DOI:** 10.3390/molecules27030846

**Published:** 2022-01-27

**Authors:** Guoli Chang, Chenggang Cai, Yannan Xiang, Xiangjun Fang, Hailong Yang

**Affiliations:** 1 School of Biological and Chemical Engineering, Zhejiang University of Science and Technology, Hangzhou 310023, China; 212003817003@zust.edu.cn (G.C.); 212103817019@zust.edu.cn (Y.X.); 2 Institute of Food Science, Zhejiang Academy of Agricultural Sciences, Hangzhou 310023, China; fangxj@zaas.ac.cn; 3 College of Life and Environmental Science, Wenzhou University, Wenzhou 325035, China

**Keywords:** *Myrica rubra* residue, diabetes management, polyphenols, α-Glucosidase inhibition, HPLC

## Abstract

*Myrica rubra* pomace accounts for 20% of the fruit’s weight that is not utilized when it is juiced. The pomace contains bioactive phenolic substances such as anthocyanins and flavonoids. To improve the utilization value of *Myrica rubra* pomace, an optimized extraction method for the residual polyphenols was developed using response surface methodology (RSM). The resulting extract was analyzed by high performance liquid chromatography (HPLC), and the in vitro hypoglycemic activity and antioxidant activity of the polyphenolic compounds obtained were also investigated. The optimum extraction conditions (yielding 24.37 mg·g^−1^ total polyphenols content) were: extraction temperature 60 °C, ultrasonic power 270 W, ethanol concentration 53%, extraction time 57 min, and solid to liquid ratio 1:34. Four polyphenolic compounds were identified in the pomace extract by HPLC: myricitrin, cyanidin-*O*-glucoside, hyperoside, and quercitrin. DPPH and hydroxyl radical scavenging tests showed that the *Myrica rubra* polyphenols extract had strong antioxidant abilities. It is evident that the residual polyphenols present in *Myrica rubra* pomace have strong hypoglycemic activity and the juiced fruits can be further exploited for medicinal purposes.

## 1. Introduction

Diabetes mellitus is a chronic metabolic disease characterized by hyperglycemia, usually caused by insufficient insulin secretion or impaired insulin action, accompanied by disorders in the metabolism of three major nutrients: sugar, fat, and protein [1]. Diabetes can cause damage and lesions in many organs of the body, including the eyes, kidneys, blood vessels, heart, and brain, and there are more than 100 kinds of complications [2], which seriously affect the daily life and physical and mental health of patients. According to the International Diabetes Federation (IDF), the prevalence of adult diabetes globally reached 9.3% in 2019, 438 million patients. China accounts for the largest grouping of diabetics, about 116 million. By 2045, nearly 700 million adults are projected to suffer from diabetes, worldwide [3]. Currently, approximately 90% of the patients clinically diagnosed with diabetes mellitus suffer from type II [4].

Controlling blood glucose levels is an effective way to reduce the effects of diabetes and the complications caused by hyperglycemia [5]. Most of the current drug-therapy glucose-lowering mechanisms for diabetes include targeting pancreatic β-cells, reducing pancreatic β-cell apoptosis, promoting β-cell proliferation and regeneration, and promoting insulin secretion. Other therapeutic approaches to managing diabetes include: increasing the number of insulin receptors or increasing their sensitivity to insulin, reducing hepatic glucose output, promoting sugar utilization by surrounding tissues and target organs, scavenging free radicals, reducing the expression of inflammatory factors and protecting pancreatic β-cells from inflammatory response damage, improving microcirculation, and inhibiting the activity of key enzymes, such as α-glucosidase and α-amylase [6]. At present, improving postprandial hyperglycemia is a key strategy for the treatment of diabetes and its complications. α-glucosidase inhibitors, such as acarbose, miglitol tablets, and voglibose, mainly delay the absorption of glucose in the body and reduce postprandial hyperglycemia by inhibiting the activity of α-glucosidase [7]. However, long-term administration of these hypoglycemic drugs can lead to serious gastrointestinal adverse effects, such as abdominal discomfort, diarrhea, and flatulence [8,9]. Therefore, many pharmaceutical developers are actively seeking safe, effective, and non-toxic alternatives. As an alternative to drug therapies, treating or controlling diabetes and preventing or delaying the occurrence of complications related to diabetes is possible by modifying diets and taking herbal medicines [10,11,12].

*Myrica rubra* (MR) is an endemic fruit in China that is cultivated in Zhejiang, Fujian, and Jiangsu provinces [13,14]. *M. rubra* contains bioactive substances, such as anthocyanins [15], flavonoids [16], and terpenoids [17], that can display free-radical scavenging antioxidant [18], anti-inflammatory [19], lipid improvement [20], cancer prevention [21], and antibacterial [22] activities. *M. rubra* can be consumed directly or processed into juices, fruit wines, fruit vinegars, beverages, etc. The pomace (residue) from juice extraction is mostly discarded as solid waste, leading to environmental pollution. It is also extremely wasteful because the residue is rich in polyphenols content, and it may also contain significant amounts of the bioactive ingredients; therefore, further study of *M. rubra* pomace is critically needed.

In this study, extraction optimization of active ingredients from *M. rubra* pomace by single-factor and RSM. The active ingredients in the extract were separated and identified by HPLC, then the in vitro antioxidant effect was also evaluated by the scavenging ability of 1,1-diphenyl-2-picylhydrazyl (DPPH) radicals and hydroxyl radicals and the in vitro hypoglycaemic effect was assessed by the α-glucosidase inhibition assay. The results will be useful for further high-valued application of *Myrica rubra* pomace as a material to extract active ingredients and estimate their activities.

## 2. Results

### 2.1. Single Factor Experimental Results

#### 2.1.1. Effect of Sonication Power on Total Polyphenols Content

**Figure 1:**
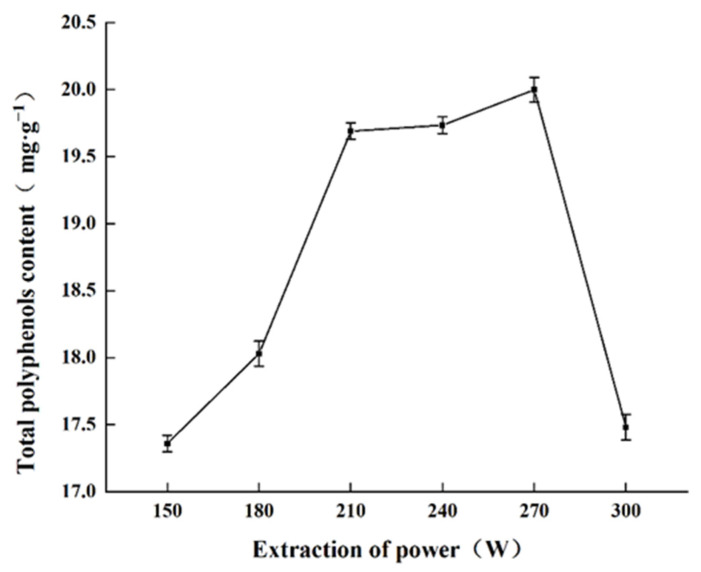
Effect of sonication power on the total polyphenols content obtained.

The effect of sonication power on the total polyphenols content from MR powder extraction is shown as Figure 1. Total polyphenols content obtained increased from 150 W to 270 W, with maximal extraction efficiency at 270 W (19.99 mg·g^−1^ obtained). Continuing to increase the ultrasonication power led to reduced extraction efficiency of polyphenols, mainly because the higher power created more cavitation bubbles [23]. Too many cavitation bubbles affects the propagation of ultrasound [24], which decreases overall extraction efficacy. Additionally, anthocyanin is susceptible to degradation when the sonication power is too high. Considering the extraction performance, anthocyanin preservation, and energy savings, 270 W was selected as the optimal ultrasonic power setting.

#### 2.1.2. Effect of Extraction Time on Total Polyphenols Content

**Figure 2:**
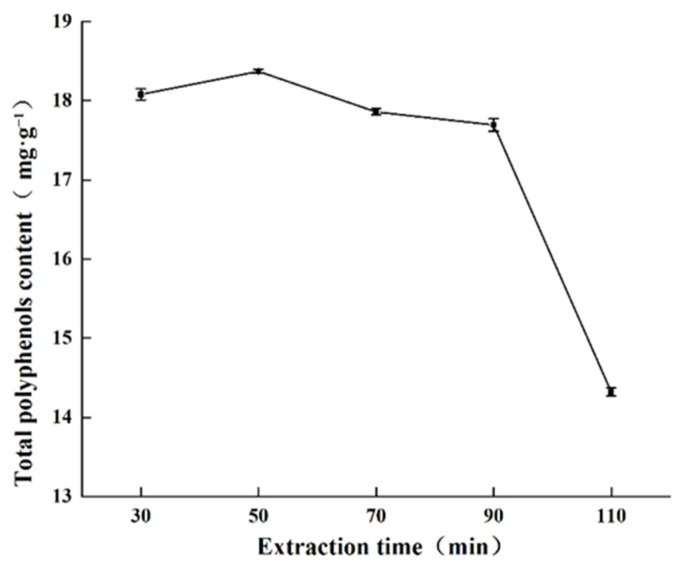
Effect of extraction time on total polyphenols content obtained.

The relationship between extraction time and total polyphenols content is shown in Figure 2. Total polyphenols content did not fluctuate with time until the 90 min mark where the total polyphenols content decreased sharply. Prolonged ultrasonic treatment, which heats up the extraction mixture, may have accelerated the oxidation and destruction polyphenols, resulting in loss of total polyphenols extraction [25]. Therefore, 50 min was selected as the optimal extraction time from this study.

#### 2.1.3. Effect of Solid to Liquid Ratio on Total Polyphenols Content

**Figure 3:**
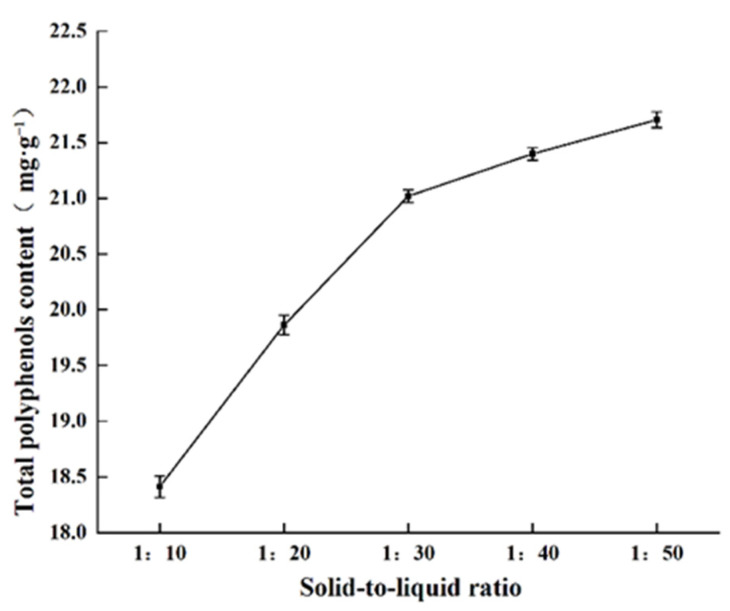
Effect of solid to liquid ratio on the total polyphenols content obtained.

The effects of various solid to liquid ratios on polyphenols extraction are shown in Figure 3. With increasing solid to liquid ratios, the total phenolic content gradually increased. When the solid to liquid ratio was 1:30, the total polyphenols content obtained was 21.02 mg·g^−1^. Further increasing the solid to liquid ratio to 1:50 led to 21.71 mg·g^−1^ total polyphenols extraction. While increasing the amount of solvent increased total polyphenols content extraction, minimizing the amount of solvent required for extraction aids in process efficiency because the extraction solvent must ultimately be removed. Therefore, solvent usage should be minimized to reduce the extraction cost from both a material and time perspective. Considering the decreasing gains from further increasing the amount of extraction solvent, the solid to liquid ratio of 1:30 was selected for further study.

#### 2.1.4. Effect of Ethanol Concentration on Total Polyphenols Content

**Figure 4:**
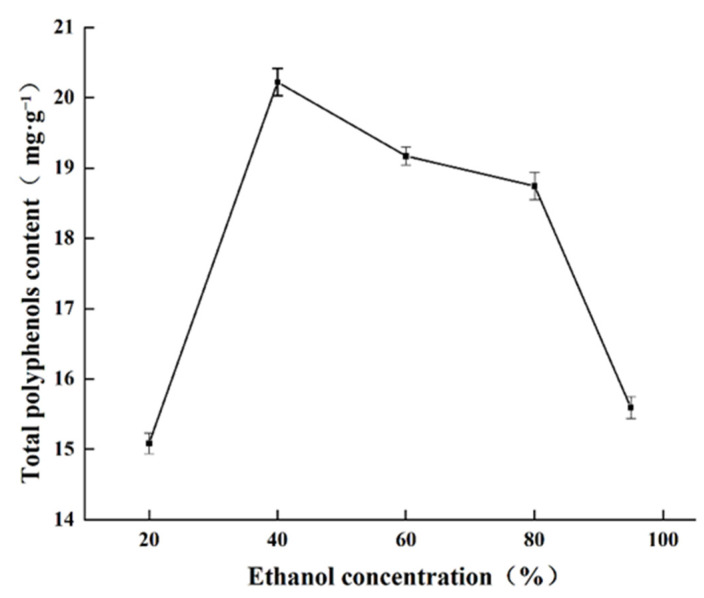
Effect of ethanol concentration on total polyphenols content obtained.

The effects of different ethanol concentrations on polyphenols extraction are shown in Figure 4. Extraction efficiency increased continuously as the percent ethanol increased, reaching a maximum of 20.22 mg·g^−1^ with 40% ethanol. Thereafter, further increasing the ethanol concentration decreased total polyphenols extraction. Phenolic compounds are mainly distributed in the cytosol of plant vesicles. Water and low concentrations of ethanol can enter the cells, but high concentrations of ethanol can lead to denaturation of plant proteins and prevent the solubilization of polyphenols, thus affecting the extraction efficiency [26]. Therefore, 40% aqueous ethanol was selected as the optimal extraction solvent.

### 2.2. Response Surface Modelling

#### 2.2.1. Analysis of the Response Surface Model

The MR powder extraction conditions were optimized using different combinations of variables according to the central composite design model in Table 1. To evaluate the applicability of the optimized model, regression analysis and analysis of variance (ANOVA) tests were performed.

Design-expert software was used for quadratic regression fitting of the data in Table 1. The regression equation for total polyphenols content obtained was: total polyphenols content (mg·g^−1^) = 22.72 + 2.48 A + 0.42 B + 1.02 B + 0.44 AB + 0.77 AC − 0.23 BC − 2.19 A^2^ − 0.92 B^2^ − 1.93 C^2^.

The RSM test was performed on the experimental data and the applicability of the model was analyzed using linear regression and ANOVA (Table 2).

As seen in Table 2, the regression model obtained from the results of this experiment were significant (*p* = 0.0034 < 0.005) and the misfit error was not significant (*p* = 0.1996 > 0.05), which indicates that the unknown factors have less influence on the test results. The residuals were mainly caused by random errors, and the test results fit the model well [27]. The adjustment coefficient R^2^Adj in the model was 79.74%, indicating that 79.74% of the variation in response values could be explained by the model. The correlation coefficient R^2^ was 89.34%, and the signal-to-noise ratio was 8.981 > 4, indicating that the model was highly credible and the model could be used for analysis and prediction. Ranking the importance of primary factors for polyphenols extraction from MR powder: ethanol concentration > solid-liquid ratio > time.

#### 2.2.2. Interactions of Different Experimental Factors on the Response Variables

**Figure 5:**
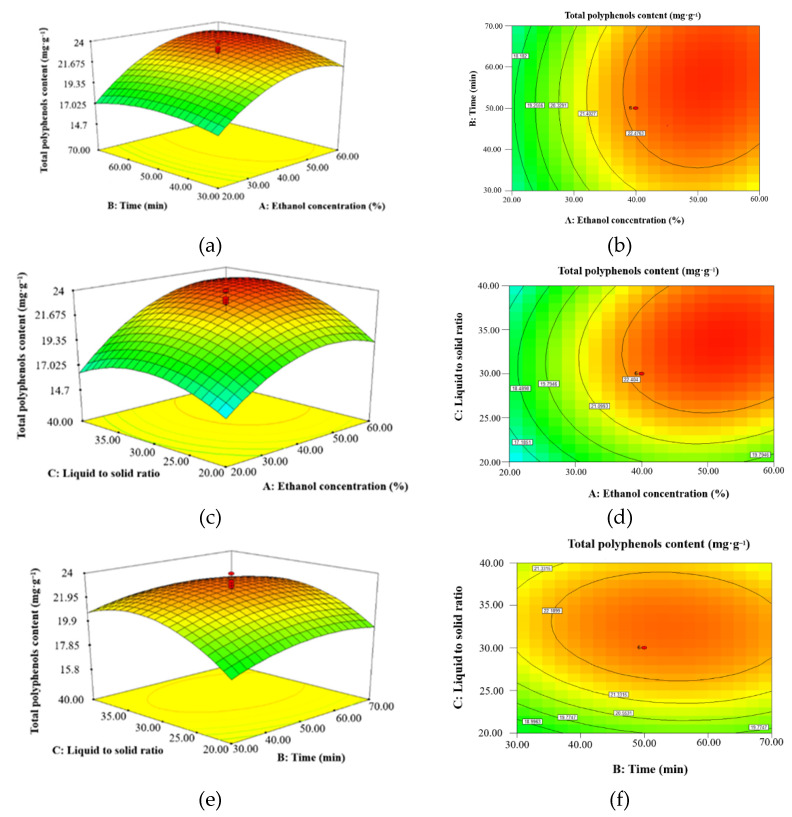
Response surface plots on total polyphenols extraction: (**a**,**b**) ethanol concentration and extraction time, (**c**,**d**) ethanol concentration and liquid to solid ratio, and (**e**,**f**) liquid to solid ratio and extraction time.

The response surface graph is a three-dimensional spatial surface graph of the response factors for each experimental factor that can visually depict the interaction between each experimental factor. The contour shape reflects the strength of the interaction: the more elliptical the contour shape, the stronger the interaction; and the rounder the contour shape, the weaker the interaction [28]. To better visualize the interaction of different experimental variables on polyphenols extraction, three response and contour plots were developed (Figure 5). The steeper the response surface and the stronger the contour lines, the more significant the influence of each factor is on the response value. The extreme values exist in the selected range, which is the highest point of the response surface and the center of the minimum ellipse of the contour line.

Figure 5a or Figure 5b shows the effect of ethanol concentration on the extraction content: the highest polyphenols extraction was achieved with 50% aqueous ethanol, and the quantity of polyphenols extracted decreased with further increasing the ethanol concentration. As the ethanol concentration increased, more lipid-soluble and alcohol-soluble components reduced tissue permeability, thus decreasing the efficiency of polyphenols extraction [29,30].

From Figure 5c or Figure 5d, the larger the liquid to solid ratio, the larger the response value, indicating that the liquid to solid ratio has a large effect on the response value. As the solvent dosage increased, the effective contact area between the solvent and the substance increased, and the solute concentration difference became larger, which facilitated dissolution in the target molecules and promoted polyphenols extraction [31]. As noted in Section 2.1.4, polyphenols extraction decreased with increasing ethanol volume [32].

Figure 5e or Figure 5f show that there is an optimal sonication time for polyphenols extraction from MR powder, because excessive ultrasonic treatment leads to decomposition and oxidation of the extracted polyphenols [33].

#### 2.2.3. Validation of Optimal Conditions

From the RSM study, the optimal extraction conditions would be: ultrasonic power 270 W, ethanol concentration 53.37%, extraction time 56.84 min, and solid to liquid ratio 1: 33.79. Under these conditions, predicted maximum polyphenols extraction from MR powder would be 23.82 mg·g^−1^. To facilitate a practical study, the parameters were rounded to: solid to liquid ratio of 1:34 (g·mL^−1^), ultrasonic time of 57 min, and 53% aqueous ethanol. Under these conditions, 24.37 mg·g^−1^ of polyphenols extraction was achieved, which was similar to the theoretical value. Therefore, the response surface optimization method can be used to optimize the extraction process of polyphenols from MR powder.

### 2.3. Antioxidant Activity of the Polyphenols Extract from MR Powder

**Figure 6:**
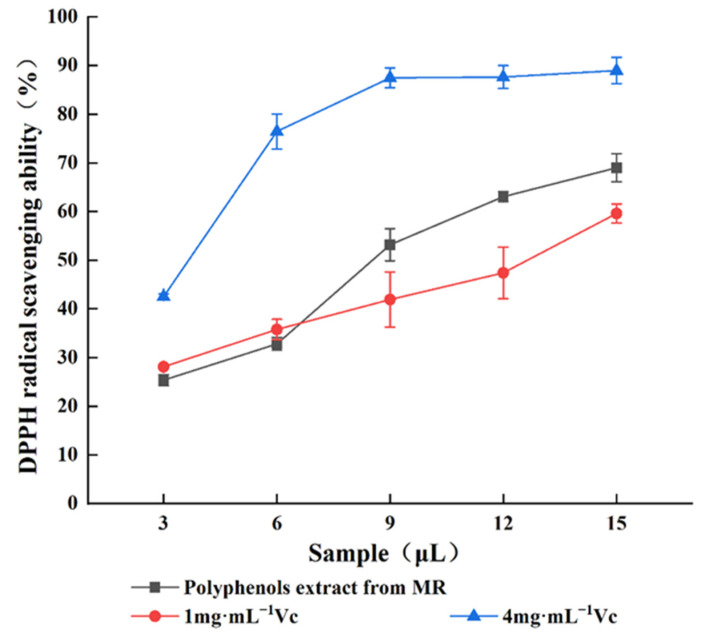
DPPH radical scavenging ability of polyphenols and Vc from MR powder extract.

**Figure 7:**
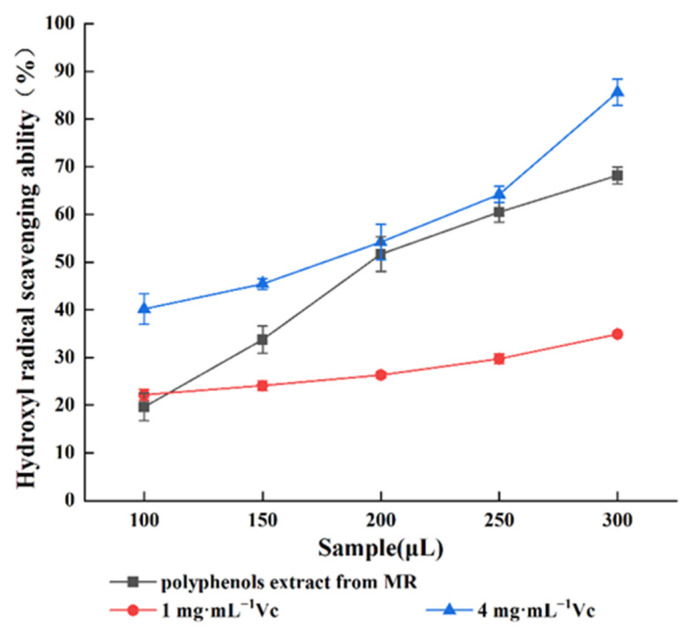
Scavenging ability of polyphenols and Vc hydroxyl radical in MR powder extract.

The antioxidant activities of MR powder extract were expressed in terms of DPPH free radical scavenging capacity (Figure 6) and hydroxyl free radical scavenging capacity (Figure 7).

As can be seen in Figure 6, the scavenging effect of DPPH radicals by the MR powder extract gradually increased (25.38% to 69.03%) with increasing sample dosage from 3 to 15 μL, showing a dose-dependent relationship. Compared with the control Vc, the scavenging ability of M. rubra residue polyphenols extract ranged between 1 mg·mL^−1^ Vc and 4 mg·mL^−1^ Vc. A strong DPPH radical scavenging effect was observed with even a small amount of MR powder extract, which indicates that the polyphenols in M. rubra present strong antioxidant ability.

As shown in Figure 7, MR powder polyphenols extract and Vc displayed strong HO scavenging ability in a dose-dependent relationship. When 300 μL was the selected dose, the hydroxyl radical scavenging ability was 34.92% for 1 mg·mL^−1^ Vc, 68.19% for MR powder polyphenols extract, and 85.62% for 4 mg·mL^−1^ Vc. The hydroxyl radical scavenging ability of MR powder polyphenols extract was between 1 mg·mL^−1^ Vc and 4 mg·mL^−1^ Vc, and showed an extremely strong increasing trend with increasing sample dosage.

### 2.4. Liquid Chromatography Analysis

**Figure 8:**
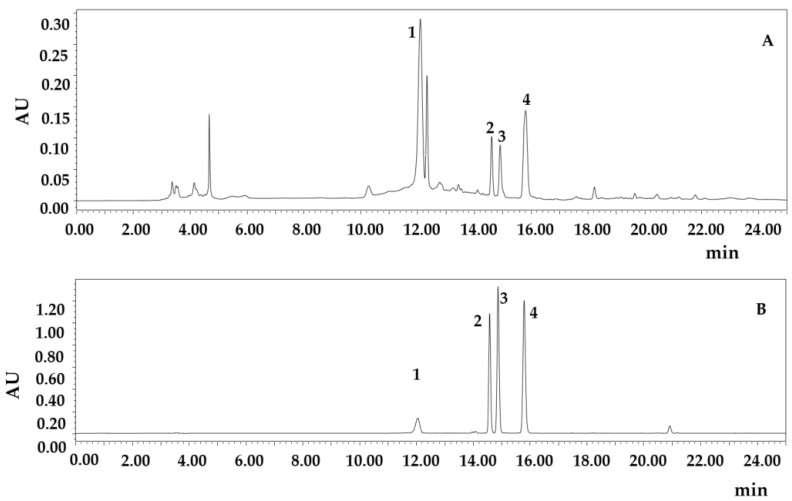
MR powder extract (**A**) and HPLC chromatograms of the reference substances (**B**). 1. Cyanidin-3-*O*-glucoside; 2. Myricitrin; 3. Hyperoside; and 4. Quercitrin.

In order to analysis the chemicals in MR powder extract, both the MR powder extract and the reference substances of Cyanidin-3-*O*-glucoside, Myricitrin, Hyperoside and Quercitrin were analyzed by HPLC, the results were shown in Figrue 8A and Figure 8B.

**Figure A1:**
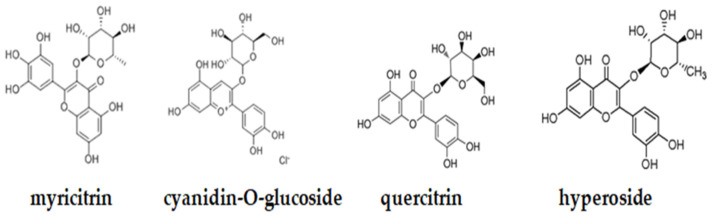
Molecular structure of four hypoglycaemic components of *Myrica rubra* pomace.

The maximum absorption wavelength of cyanidin-*O*-glucoside was 279.7 nm, that of myricitrin was 352.5 nm, that of hyperoside was 357.3 nm, and that of quercitrin was 355 nm. After a full wavelength scanning of the standards, the peak areas of the controls were read at the corresponding maximum absorption wavelengths, and the standard curves were plotted using concentration as the horizontal coordinate (x) and the peak area (y) as the vertical coordinate. Linear regression equations of cyanidin-o-glucoside, myricitrin, hyperoside and quercitrin were y = 16051x − 15738 (R^2^ = 0.9993), y = 21341x − 149304 (R² = 0.9886), y = 27830x − 139116 (R² = 0.9954), and y = 27394x − 16759, (R² = 0.998), respectively. Having calibrated with standards, the concentrations of cyanidin-*O*-glucoside, myricitrin, hyperoside, and quercitrin obtained from the MR powder were 322.22 mg·L^−1^, 23.00 mg·L^−1^, 17.81 mg·L^−1^, and 24.35 mg·L^−1^, respectively. The structure of cyanidin-o-glucoside, myricitrin, hyperoside and quercitrin were listed as Appendix A (Figure A1).

### 2.5. Analysis of α-Glucosidase Inhibition Experiments

**Figure 9:**
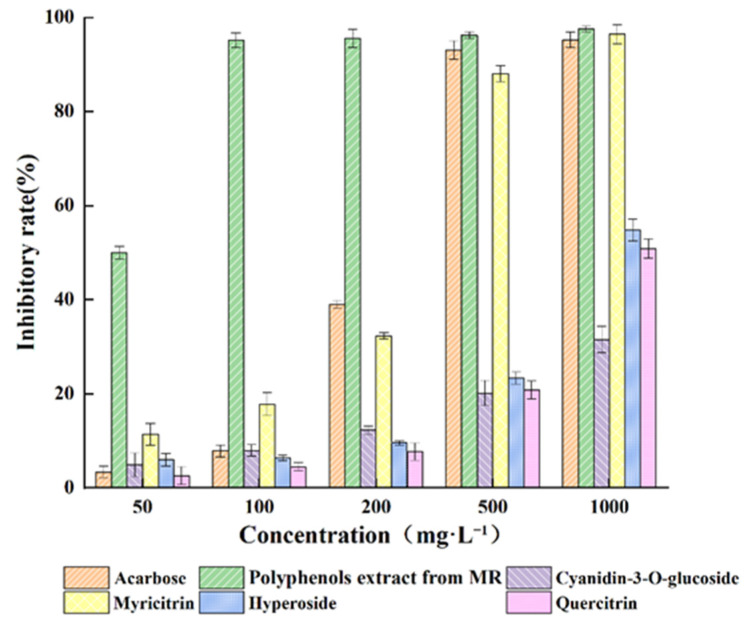
Inhibitory effects of MR powder extract on α-glucosidase activity.

As can be seen in Figure 9, the inhibition rate for each substance on α-glucosidase increased with increasing dosage. At the final concentration of 1000 mg·L^−1^, the positive control (acarbose) inhibited α-glucosidase by 95.24%, the polyphenols extract from MR powder inhibited α-glucosidase by 97.57%, cyanidin-*O*-glucoside, myricitrin, hyperoside, and quercitrin inhibited α-glucosidase by 31.53%, 96.408%, 54.80%, and 50.88%, respectively. The IC_50_ values of acarbose, polyphenols from MR powder extract, cyanidin-*O*-glucoside, myricitrin, hyperoside, and quercitrin were 231.41, 39.04, 3071.33, 221.33, 1056.20, and 1096.97 mg·L^−1^, respectively. Thus, the MR powder extract demonstrated strong hypoglycemic effects, and the order of hypoglycemic strength of the active ingredients was myricitrin > hyperoside > quercitrin> cyanidin-*O*-glucoside. Additionally, the hypoglycemic activity of the crude MR powder extract showed a positive correlation with the four flavonoid glycosides.

## 3. Materials and Methods

### 3.1. Materials

Fresh *Myrica rubra* pomace was provided by a farmer located in Ningbo, Zhejiang Province, China. The pomace was dried at 40 °C overnight, then the residue was ground and filtered through a 40-mesh screen and stored at −20 °C until use (MR powder).

### 3.2. Chemicals and Solvents

1,1-diphenyl-2-picylhydrazyl (DPPH), Folin-Ciocalteu reagent, ascorbic acid (AA), rutin, α-glucosidase, gallic acid (GA), cyanidin-*O*-glucoside, myricitrin, hyperoside, and quercitrin were purchased from Sigma Chemical Co. (St. Louis, MO, USA). All other chemicals, solvents, and reagents were analytical grade and obtained from Aladdin Chemical Corp. Ltd. (Shanghai, China).

### 3.3. Extraction Procedure

MR powder (500 mg) was accurately weighed and placed in stoppered glass test tubes. When ready, aqueous ethanol (various volumes and concentrations) was added and the stoppered tubes were placed in an ultrasonic water bath (KQ-400KDB, Kun Shan Ultrasonic Instruments Co., Ltd., Kunshan, China). After ultrasonic treatment, the tubes were centrifuged at 3000 rpm for 5 min and the supernatant was collected for polyphenols content determination.

### 3.4. Experimental Design and Analytical Methods

#### 3.4.1. Single-Factor Extraction Experiments for Polyphenols MR Powder

The effects of different ultrasonic powers (150, 180, 210, 240, 270, and 300 W), aqueous ethanol concentrations (20%, 40%, 60%, 80%, and 95%), extraction time (30, 50, 70, 90, and 110 min), and solid-liquid ratios (1:10, 1:20, 1:30, 1:40, and 1:50) on the polyphenols content in the extracts were investigated by conducting single-factor tests: in the development of one of the factors, the parameters of the other factors were taken as the middle value of the experimental design gradient, i.e., ultrasonic power of 240 W, aqueous ethanol concentration of 60%, extraction time of 70 min, and solid-liquid ratios 1:30. Factors with significant effects on polyphenols content were selected for further study using response surface tests, which were repeated in triplicate.

#### 3.4.2. Response Surface Experiments for Polyphenols Extraction from MR Powder

Based on the single-factor tests, the three factors of ethanol concentration (A), extraction time (B), and solid-liquid ratio (C) were selected as the independent variables to optimize the polyphenols extraction content (Y) by central composite design experiments using Design expert 7.0 software (Stat-Ease, Inc., Minneapolis, MN, USA). The experimental data were fitted and processed, and the test factors and levels are shown in Table 3.

#### 3.4.3. Determination of Gallic Acid (GA) Content

GA content was analyzed according to method of Francois [34] with slight modifications: extract solution (0.5 mL), distilled water (9.5 mL), and Folin-Ciocalteu reagent (1 mol·L^−^^1^, 1 mL) were sequentially added into a 25 mL volumetric flask and mixed thoroughly. Then, 7% sodium carbonate solution (5 mL) was added. The mixed solution was allowed to sit at room temperature for 1 h in the dark. Then, distilled water was added to the volumetric flask of 25 mL and the absorbance was analyzed at 750 nm. GA standard solutions of 100, 200, 300, 400, and 500 mg·L^−1^ were prepared and utilized to generate a calibration curve. The regression linear equation obtained was: y = 0.0014x + 0.062, R² = 0.9946. The concentration (mg·g^−1^) was expressed as ratio of the determined results to fresh weight (FW). All samples were analyzed in triplicate.

#### 3.4.4. Determination of Total Flavonoids

The total flavonoid content was determined according to Hossain [35] with slight modifications. Seven 25 mL volumetric flasks were used to prepare 0.02, 0.04, 0.06, 0.08, 0.1, and 0.12 mg·mL^−1^ rutin solutions using 30% ethanol as solvent. The above rutin standard solution (5 mL) was dispensed into seven stoppered test tubes; thereafter, 5% aqueous NaNO_2_ (0.3 mL) was added to the serial dilutions and thoroughly mixed for 6 min. Then, 10% aqueous Al(NO_3_)_3_ (0.3 mL) was added to the serial dilutions and thoroughly mixed for 6 min. Finally, the reaction was quenched with 1M aqueous NaOH (4 mL) and distilled water (0.4 mL). After 15 min, the absorbance value was measured at 510 nm. The regression linear equation obtained for the rutin standard curve, utilized as a representative for total flavonoid content, was: y = 0.1502x + 0.0603, R^2^ = 0.9978. Samples of crude MR powder extract (2 mL) were similarly processed to determine flavonoid content. All experiments were performed in triplicate.

#### 3.4.5. Determination of Total Anthocyanin

The total anthocyanin content was determined by the pH differential method using the method of Wang [36] with slight modifications. The sample solution (0.5 mL) was pipetted into a 10 mL tube and supplemented with 9.5 mL of pH 1.0 (0.2 M HCl: 0.2 M KCl = 25:67 (*V*:*V*)) and pH 4.5 (0.2 M HOAc: 0.2 M NaOAc = 63:37(*V*:*V*)) buffers, respectively, and equilibrated for 30 min with distilled water as a control. The absorbance values at 510 nm and 700 nm were measured with distilled water as control, and the experiments were performed in triplicate.

(1) $$\mathrm{Total}\;\mathrm{absorbance}\;A=\frac{A510-A700}{A^{\prime }510-A^{\prime }700}\;$$

(2) $$\mathrm{Anthocyanin}\;\mathrm{content}\;\left(\mathrm{mg}\cdot L^{-1}\right)=\frac{A\times \;1000\times \;\mathrm{MW}\;\times \;\mathrm{dilution}\;\mathrm{ratio}\;\times V}{L\varepsilon \times m}=\frac{A\times \;1000\times \;449.2\;\times \;20}{26900}\;$$

where A510, A700 are absorbance values under pH 1.0; A’510 and A’700 are the absorbance values at pH 4.5; L is 1, the width of the colorimetric cup; ε, 26900, the molar absorbance coefficient of cyanidin-3-*O*-glucoside in bayberry; 449.2 is the relative molecular weight of cyanidin-3-*O*-glucoside; V is 10 mL; and m is sample mass.

#### 3.4.6. Total Phenolic Content Calculation

Three representative polyphenols with highest content in the crude MR powder extract were selected: anthocyanins, flavonoids, and GA. Total phenolic content of the MR powder extract was the summation of these three measured substance values [37].

#### 3.4.7. DPPH Scavenging Capacity

The method employed by Li [38] was modified by adding 3, 6, 9, 12, and 15 μL of sample to 2 mL of deionized water, 4 mL of 0.1 mmol·L^−1^ DPPH-methanol solution, and 450 μL of 50 mmol·L^−1^ Tris-HCl buffer (pH 7.4). The reaction was carried out at 25 °C for 30 min and the absorbance was measured at 517 nm using deionized water as the reference solution and 1 mg·mL^−1^ ascorbic acid (Vitamin C, Vc) and 4 mg·mL^−1^ Vc as the positive control.

(3) $$\mathrm{DPPH}\;\mathrm{radical}\;\mathrm{scavenging}\;\mathrm{ability}\;\left(\%\right)=\frac{A_{0}-\left(A_{1}-A_{2}\right)}{A_{0}}\times 100\;$$

where A_0_ is the absorbance of blank control solution; A_1_ is the sample absorbance; and A_2_ is the sample background absorbance without DPPH-methanol solution.

#### 3.4.8. Determination of Hydroxyl Radical Scavenging Ability

Referring to the method of Tohidi [39], 100, 150, 200, 250, and 300 μL of the samples were added to deionized water (2 mL), then 6 mM H_2_O_2_ (1.4 mL), followed by 20 mM sodium salicylate (0.6 mL) and 1.5 mM aqueous ferrous sulphate (2 mL). The reaction vessel was placed in a constant water bath at 37 °C for 1 h. Deionized water was used as the reference solution and the absorbance was measured at 562 nm using 1 mg·mL^−1^ Vc and 4 mg·mL^−1^ Vc as the positive controls.

(4) $$\mathrm{Hydroxyl}\;\mathrm{radical}\;\mathrm{scavenging}\;\mathrm{ability}\;\left(\%\right)=\frac{A_{0}-\left(A_{1}-A_{2}\right)}{A_{0}}\times 100$$

where A_0_ is the absorbance of blank control solution; A_1_ is the sample absorbance; and A_2_ is the sample background absorbance without H_2_O_2_ solution.

#### 3.4.9. Liquid Chromatography Analysis

The composition of the *M. rubra* residue polyphenols extract was analyzed using a Waters 2695 HPLC apparatus with Photodiode Array Detector 2998 (Waters, Framingham, MA, USA).

Chromatographic conditions: TechMate C18-ST column (4.6 × 250 mm, 5 μm, Techmate, Beijing, China), 100% methanol (mobile phase A) and 0.1% formic acid water (mobile phase B). Gradient elution program: 0–5 min, 20% A; 5–10 min, 20% to 60% A; 10–13 min, 60% A; 13–15 min, 60% to 80% A; 15–20 min, 80% A; 20–23 min, 80% to 20% A; 23–25 min, 20% A; flow rate, 0.8 mL·min^−1^; detection wavelength, 220~400 nm full wavelength scan; column temperature, 35 °C; and injection volume, 10 μL.

Preparation of mixed control solution: 10 mg of cyanidin-*O*-glucoside, myricitrin, hyperoside, and quercitrin were weighed precisely, placed in a 10 mL volumetric flask, mixed with methanol, and then diluted into mixed control solutions with mass concentrations of 5, 10, 20, 50, 100, and 200 mg·L^−1^, respectively.

Sample preparation: 1 mL of MR powder polyphenols extract, obtained from the best extraction conditions as determined from the response surface test experiments, was passed through a 0.45 μm syringe filter (nylon 66) and stored in the autosampler vial.

#### 3.4.10. α-Glucosidase Inhibition Experiment

The inhibitory effect of the crude MR powder extracts on α -glucosidase was determined using 4-nitro-α-d-glucopyranoside (PNPG) as a substrate. The studies for α-glucosidase inhibition followed the method of Yao [40] with slight modifications. Experiments were performed in 96-well plates. α-glucosidase (0.32 U·mL^−1^, 20 μL) and various concentrations sample solution (20 μL) were incubated in 100 μL phosphate buffer (pH 6.8) at 37 °C for 10 min, then PNPG (20 μL, 2.5 mmol·L^−1^) was added. The plate was incubated at 37 °C for 15 min. Reactions were terminated with 40 μL 0.2 M NaOH. The absorbance was measured at 405 nm. The blank and background controls were specific wells within the plate. Acarbose was used as a positive control. Inhibitor concentration of 50% (IC_50_) was determined when α-glucosidase activity was inhibited 50%. The inhibition rate of α-glucosidase and IC_50_ values of each sample were calculated as follows:

(5) $$\alpha -\mathrm{glucosidase}\;\mathrm{inhibition}\;\mathrm{rate}\;\left(\%\right)=\frac{A_{b}-\left(A_{s}-A_{0}\right)}{A_{b}}\times 100$$

where A_b_ is the absorption value of the control group; A_s_ is the absorbance value of sample group; and A_0_ is the absorption value of the background group.

#### 3.4.11. Statistical Analysis

RSM experiments were conducted according to Design expert 7.0 software (Stat-Ease, Inc., Minneapolis, MN, USA). Each experimental data point in this paper is represented as mean ± standard deviation (SD) of three independent replicates. The significant differences among means were identified by one-way analysis of variance (ANOVA) and a least-significant-difference (LSD) test at *p* < 0.05, conducted with the SPSS 20.0 software package (SPSS Inc., Chicago, IL, USA).

## 4. Conclusions

In this study, optimal polyphenols extraction from dried *M. rubra* pomace were obtained after single factor design and response surface methodology experiments. The best extraction conditions for polyphenols were: 60 °C, 270 W ultrasonic power, 53% ethanol concentration, 57 min ultrasonic time, and 1:34 solid to liquid ratio. The polyphenol content extracted was 24.37 mg·g^−1^. MR powder extract demonstrated strong radical scavenging abilities (both DPPH radicals and hydroxyl radicals), stronger than 1 mg·mL^−1^ Vc and close to 4 mg·mL^−1^ Vc under the same conditions. HPLC studies confirmed the presence of four hypoglycemic substances that could inhibit the activity of α-glucosidase were present in the MR powder extract: cyanidin-o-glucoside, myricitrin, hyperoside, and quercitrin. The IC_50_ values were compared, and the order of the hypoglycemic effect of the polyphenols from MR powder extract compared with the four substances and acarbose were MR powder extract > myricitrin > acarbose > hyperoside > quercitrin > cyanidin-*O*-glucoside.

*Myrica rubra* polyphenols demonstrate good antioxidant and hypoglycemic activities; future work will be carried out to explore the active mechanism of the extract polyphenols in vivo, and to compare the single or synergistic effects among the four identified ingredients of myricitrin, cyanidin-*O*-glucoside, hyperoside, and quercitrin. The results will be helpful for the development and utilization of *Myrica rubra* pomace resources.

## Figures and Tables

**Table 1:** Response surface test design and MR powder extraction results.

Number	Ethanol Concentration (%)	Time (min)	Solid-to-Liquid Ratio	Total Polyphenols Content (mg·g^−^^1^)
1	20 (−1)	70 (1)	1:20 (−1)	14.0994
2	40 (0)	16.36 (−1.682)	1:30 (0)	19.5022
3	40 (0)	50 (0)	1:30 (0)	23.9611
4	40 (0)	50 (0)	1:30 (0)	23.0864
5	73.64 (1.682)	50 (0)	1:30 (0)	20.1641
6	40 (0)	50 (0)	1:30 (0)	21.224
7	6.36 (−1.682)	50 (0)	1:30 (0)	14.7639
8	20 (−1)	30 (−10)	1:20 (−1)	13.8511
9	60 (1)	70 (1)	1:40 (1)	22.1833
10	40 (0)	50 (0)	1:30 (0)	23.3016
11	60 (1)	70 (1)	1:20 (−1)	19.0647
12	60 (1)	30 (−1)	1:40 (1)	21.0816
13	40 (0)	50 (0)	1: 46.82 (1.682)	20.6164
14	40 (0)	50 (0)	1: 13.18 (−1.682)	15.8053
15	20 (−1)	30 (−1)	1:40 (1)	14.795
16	20 (−1)	70 (1)	1:40 (10	12.971
17	40 (0)	50 (0)	1:30 (0)	21.5682
18	60 (1)	30 (−1)	1:20 (−1)	18.1948
19	40 (0)	50 (0)	1:30 (0)	22.8778
20	40 (0)	83.64 (1.682)	1:30 (0)	22.6394

**Table 2:** ANOVA of the central composite design.

Source	Quadratic Sum	Degrees of Freedom	Mean Square	F Value	*p* Value	Significance
		df			Prob > F	
Model	223.38	9	24.82	9.31	0.0009	**
A-Ethanol concentration	84.1	1	84.1	31.55	0.0002	**
B-Time	2.36	1	2.36	0.88	0.3693	
C-Solid-to-liquid ratio	14.17	1	14.17	5.32	0.0438	*
AB	1.57	1	1.57	0.59	0.4601	
AC	4.79	1	4.79	1.8	0.2098	
BC	0.42	1	0.42	0.16	0.6986	
A2	69.14	1	69.14	25.94	0.0005	**
B2	12.07	1	12.07	4.53	0.0592	
C2	53.47	1	53.47	20.06	0.0012	**
Residual error	26.66	10	2.67			
Lack of fit	21.07	5	4.21	3.77	0.0858	N
Pure error	5.59	5	1.12			
Total value	250.03	19				

Notice: ** high significant effect, *p* < 0.01; * significant effect, *p* < 0.05; N insignificant effect, *p* > 0.05; df, degrees of freedom, indicate number of items used; F value, a test statistic that determines whether any item in the model is associated with a response, including block and factor items; Prob, probability, used to measure evidence against the null hypothesis.

**Table 3:** Factors and level of response surface test for polyphenols extraction from MR powder.

Factors	Code	Level				
		−1.682	−1	0	1	1.682
Ethanol concentration (%)	A	6.36	20	40	60	73.64
Time (min)	B	16.36	30	50	70	83.64
Solid-to-liquid ratio	C	1:13.18	1:20	1:30	1:40	1:46.82

## Data Availability

All data are included in this manuscript.
